# Increased risk of open-angle glaucoma in non-smoking women with obstructive pattern of spirometric tests

**DOI:** 10.1038/s41598-022-21225-0

**Published:** 2022-10-08

**Authors:** Jihei Sara Lee, Yong Joon Kim, Sung Soo Kim, Sungeun Park, Wungrak Choi, Hyoung Won Bae, Chan Yun Kim

**Affiliations:** Department of Ophthalmology, Severance Hospital, Institute of Vision Research, Yonsei University College of Medicine, 50 Yonsei-ro, Seodaemun-gu, Seoul, 03722 South Korea

**Keywords:** Optic nerve diseases, Chronic obstructive pulmonary disease

## Abstract

To investigate differences in the prevalence of open-angle glaucoma (OAG) between different pulmonary function types. A population-based, cross-sectional analysis was conducted using Korean National Health and Nutrition Examination Surveys from 2008 to 2011. A total of 8941 subjects  ≥ 40 years of age were analyzed. Chronic obstructive pulmonary disease (COPD) was defined as the ratio between first second of forced expiration (FEV1) and forced vital capacity (FVC) below 70%. The prevalence of glaucoma, as defined by the International Society of Geographical and Epidemiological Ophthalmology, was the main outcome. OAG was more prevalent in women with COPD (8.0% vs. 4.8% normal, P = 0.001) compared to women with normal pulmonary function. Intraocular pressure (IOP) of women with COPD were 13.3 (0.2) mmHg (13.9 (0.1) mmHg for normal function, P = 0.182). Never-smokers were more prevalent in women with COPD and glaucoma (92.9% vs. 52.4% normal function; P < 0.001). COPD was found to increase the risk of glaucoma in women (OR 2.077, P = 0.017) and even further in non-smoking women (OR 2.711, P = 0.003). Women with COPD showed a higher glaucoma prevalence despite similar IOP in comparison to women with normal pulmonary function. Non-smoking COPD is significantly associated with open-angle glaucoma in women.

## Introduction

Open-angle glaucoma (OAG) is a chronic progressive optic neuropathy which results in retinal ganglion cell death and structural change in the optic nerves. It remains one of the leading causes of irreversible blindness worldwide^1^. As population is aging, glaucoma is becoming a growing public health concern^2^, and identifying individuals at risk for early treatment is increasingly emphasized to prevent detrimental disability. Many studies to date have identified various risk factors: intraocular pressure (IOP), age, family history, migraine and cardiovascular diseases such as hypertension, just to name a few^3,4^. Identification of risk factors has not only allowed early treatment, but a greater insight into the pathogenesis of the disease.

Pulmonary conditions have received little attention so far as a risk factor, but there has consistently been evidence for their role in increasing the risk of OAG if not in the pathogenesis of OAG. For instance, obstructive sleep apnea has been postulated to accelerate structural progression of glaucoma^5^. Pulmonary hypertension has been established as a cause for secondary increase in IOP^6^. Furthermore, poor lung function may affect the posterior orbit and increase IOP^7^. Finally, hypoxia, lung hyperinflation, pulmonary vascular abnormality as well as negative impact on the contractility of the heart from chronic obstructive pulmonary disease (COPD) all amount to varying degrees of cardiovascular diseases, such as ischemic coronary disease, arrhythmia and heart failure^8^, and vascular diseases^9^ are a well-known risk factor for OAG. However, no studies to date have conducted a thorough analysis on the association between different pulmonary functions and glaucoma prevalence, especially obstructive lung disease and glaucoma. The availability of visual field test, fundus photography and spirometry results in Korean National Health and Nutrition Examination Survey (KNHANES) between 2008 and 2011 has allowed us to perform a population-based, cross-sectional analysis on this topic. The aim of this study was to identify the association between pulmonary functions and the prevalence of OAG.

## Results

### Baseline characteristics of study population

**Figure 1:**
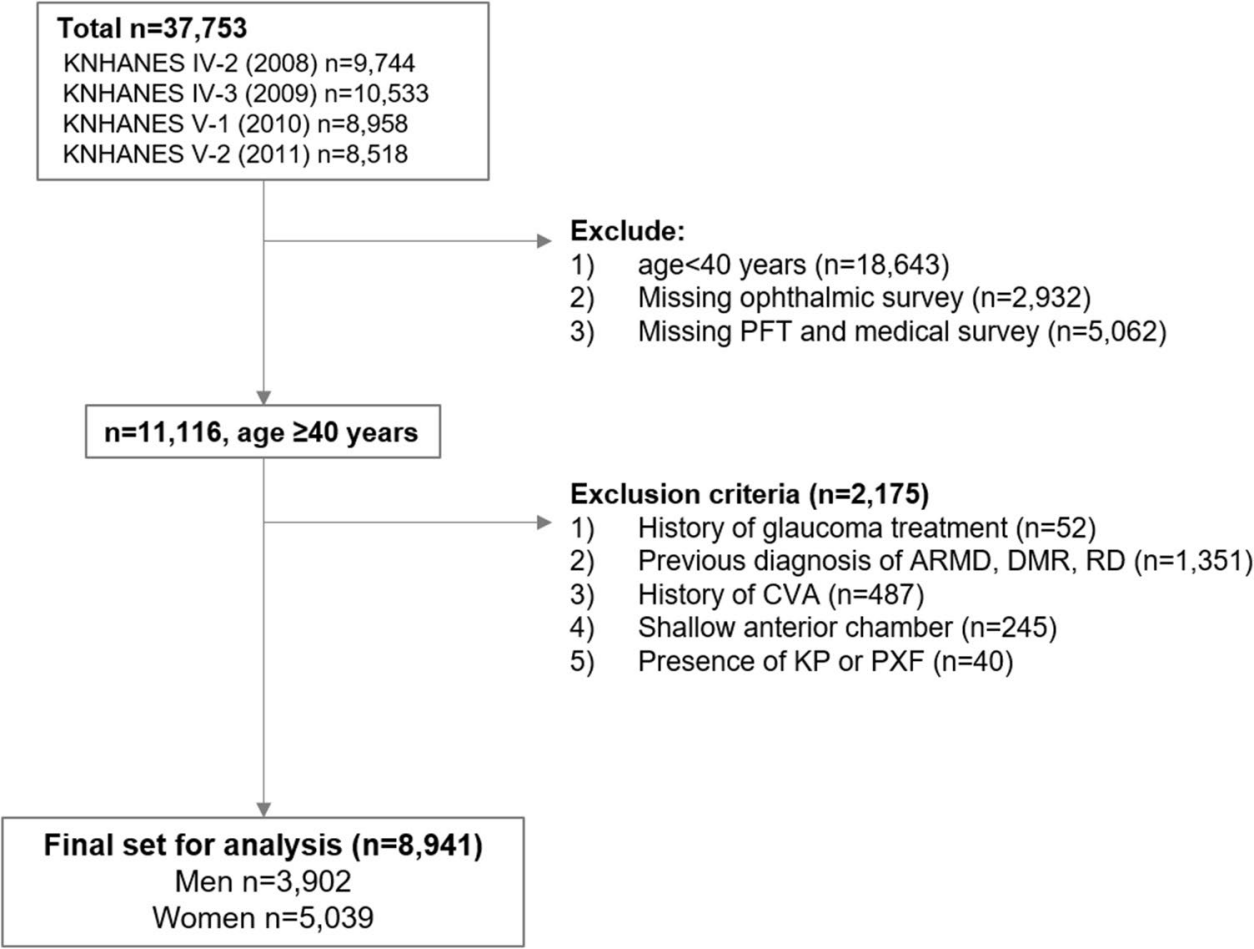
A flowchart of subject inclusion for the study. Of 37,753 subjects from Korea National Health and Nutrition Examination Survey (KNHANES), 8941 subjects were included for analysis in this study. *PFT* pulmonary function test, *ARMD* age-related macular degeneration, *DMR* diabetic retinopathy, *RD* retinal detachment, *CVA* cerebrovascular accident, *KP* keratic precipitates, *PXF* pseudoexfoliation, *IOP* intraocular pressure.

Of 37,753 subjects initially included in KNHANES from 2008 to 2011, a total of 8941 subjects (3902 men, 5039 women) were analyzed in this study (Fig. 1). Baseline characteristics of men and women according to the results of their pulmonary function test (PFT) are presented in Table 1. The obstructive group (62.6 ± 0.6 years old, men; 65.6 ± 1.0 years old, women) was generally older than the normal and restrictive groups for both men and women (restrictive 53.0 ± 0.8 years, normal 44.2 ± 0.3 years, P < 0.001 for men; restrictive 58.2 ± 1.1 years, normal 48.6 ± 0.3 years, P < 0.001 for women). While the majority of all six groups were never-smokers, the obstructive groups included higher proportions of self-reported never-smokers (73.6% vs. 51.3% normal function for men, 74.3% vs. 59.6% normal function for women) and lower proportions of ex-smokers (6.5% vs. 15.2% normal function for men; 6.9% vs. 13.0% normal function for women) than the normal groups.

**Table 1:** Baseline characteristics in men and women by pulmonary function.

	Men (n = 3902)				Women (n = 5039)			
	Normal (n = 2687)	Restrictive (n = 460)	Obstructive (n = 755)	P	Normal (n = 4282)	Restrictive (n = 469)	Obstructive (n = 288)	P
**Demographic variables**								
Age, years	44.2 (0.3)	53.0 (0.8)	62.6 (0.6)	< 0.001	48.6 (0.3)	58.2 (1.1)	65.6 (1.0)	< 0.001
BMI, kg/m^2^	24.5 (0.1)	25.3 (0.2)	23.5 (0.1)	< 0.001	24.0 (0.1)	24.4 (0.2)	23.5 (0.3)	0.631
**Health-related behavior**								
Smoking								
Never-smoker	1378 (51.3)	268 (58.3)	556 (73.6)	< 0.001	2550 (59.6)	286 (61.0)	214 (74.3)	< 0.001
Ex-smoker	408 (15.2)	51 (11.1)	49 (6.5)		557 (13.0)	56 (11.9)	20 (6.9)	
Current smoker	901 (33.5)	141 (30.7)	150 (19.9)		1175 (27.4)	127 (27.1)	54 (18.8)	
Vigorous exercise								
0–2 days/week	2266 (84.3)	380 (82.6)	585 (77.5)	< 0.001	3540 (82.7)	397 (84.6)	222 (77.1)	0.002
3–5 days/week	278 (10.3)	64 (13.9)	149 (19.7)		526 (12.3)	62 (13.2)	52 (18.1)	
6–7 days/week	142 (5.3)	16 (3.5)	13 (1.7)		208 (4.9)	10 (2.1)	10 (3.5)	
**Medical comorbidities**								
Hypertension	1048 (39.0)	238 (51.7)	382 (50.6)	< 0.001	1356 (31.7)	249 (53.1)	136 (47.2)	< 0.001
Diabetes mellitus	277 (10.3)	88 (19.1)	125 (16.6)	< 0.001	304 (7.1)	86 (18.3)	25 (8.7)	< 0.001
Dyslipidemia	382 (14.2)	69 (15.0)	102 (13.5)	0.648	768 (17.9)	112 (23.9)	68 (23.6)	0.017
**Refractive status**								
Hyperopia (> 1.00 D)	547 (20.4)	159 (34.6)	327 (43.3)	< 0.001	1150 (26.9)	169 (36.0)	119 (41.3)	< 0.001
Emmetropia	1466 (54.6)	211 (45.9)	338 (44.8)		2323 (54.3)	219 (46.7)	132 (45.8)	
Myopia								
Low (− 1.00 to − 2.99 D)	410 (15.3)	54 (11.7)	64 (8.5)		532 (12.4)	60 (12.8)	31 (10.8)	
Intermediate (− 3.00 to − 5.99 D)	203 (7.6)	28 (6.1)	18 (2.4)		198 (4.6)	18 (3.8)	3 (1.0)	
High (< − 5.99 D)	61 (2.2)	8 (1.7)	8 (1.1)		79 (1.8)	3 (0.6)	3 (1.0)	
IOP, mmHg	14.2 (0.8)	14.7 (0.2)	14.0 (0.1)	0.520	13.9 (0.1)	14.2 (0.2)	13.3 (0.2)	0.182
**Glaucoma**	217 (8.1)	41 (8.9)	63 (8.3)	0.966	204 (4.8)	30 (6.4)	23 (8.0)	**0.001**

*BMI* body mass index, *IOP* intraocular pressure.

Significant values are in bold.

A P value < 0.05 was considered statistically significant.

### Comparison of IOP and OAG prevalence

**Figure 2:**
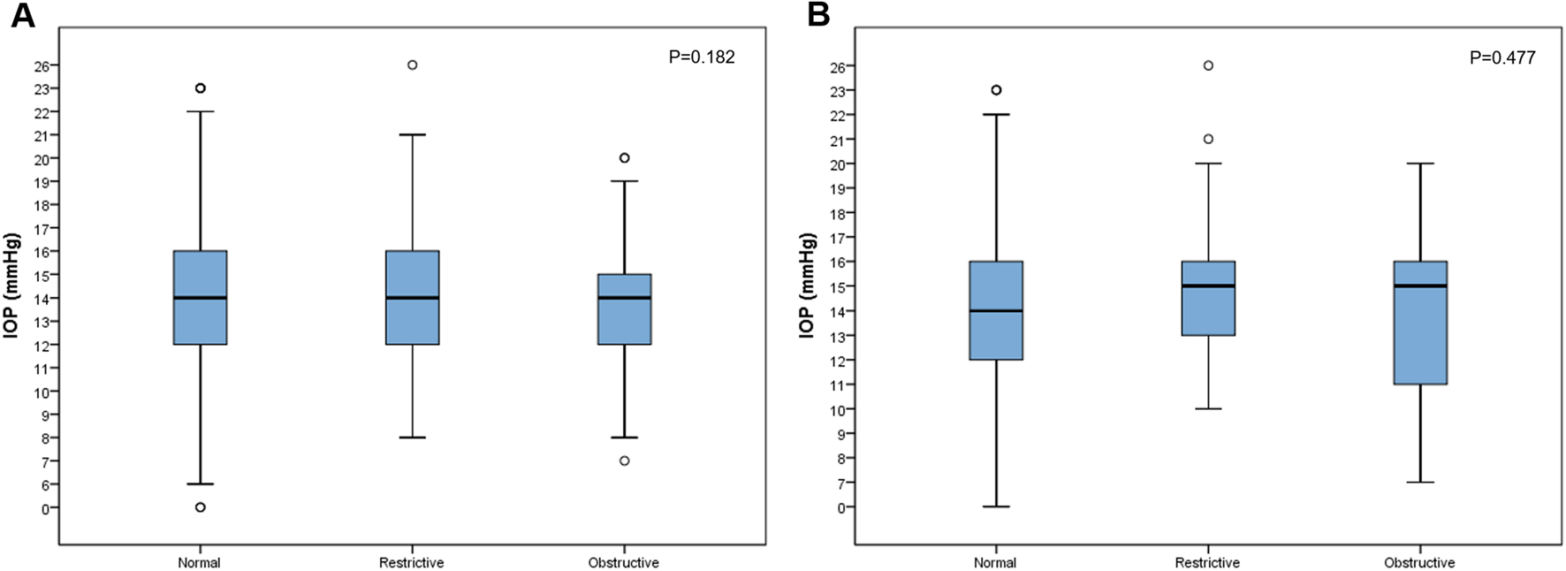
Boxplot for comparison of IOP depending on pulmonary function in women (**A**) and among women with glaucoma (**B**). Comparisons of weighted estimates of average of IOP between the three groups showed that IOP was not different in women of different pulmonary function types (**A**, P = 0.182). When women with glaucoma were separately analyzed, no statistically significant difference was noted (**B**, P = 0.477).

When average IOP was compared across the three groups, neither women nor men showed significant differences. Figure 2A illustrates the distribution of IOP for women. Women with COPD showed significantly lower IOP in comparison to women with normal function (P = 0.011). When women with glaucoma were singled out from each group and compared again, the weighted average between the three groups did not show a statistically significant difference, either (Fig. 2B). However, when the prevalence of OAG was calculated, men showed similar distribution of the disease across the three groups (8.3% obstructive vs. 8.1% normal; P = 0.966). In women, on the contrary, a significantly higher prevalence was noted in the obstructive group (8.0% vs. 4.8% normal; P = 0.001) in comparison to the other two groups (Table 1). According to our data review, none of the women diagnosed with glaucoma were on COPD medication. Among men with glaucoma, one was on COPD medication and another man had a previous history of COPD treatment.

### Prevalence of never-smokers in women with glaucoma

**Figure 3:**
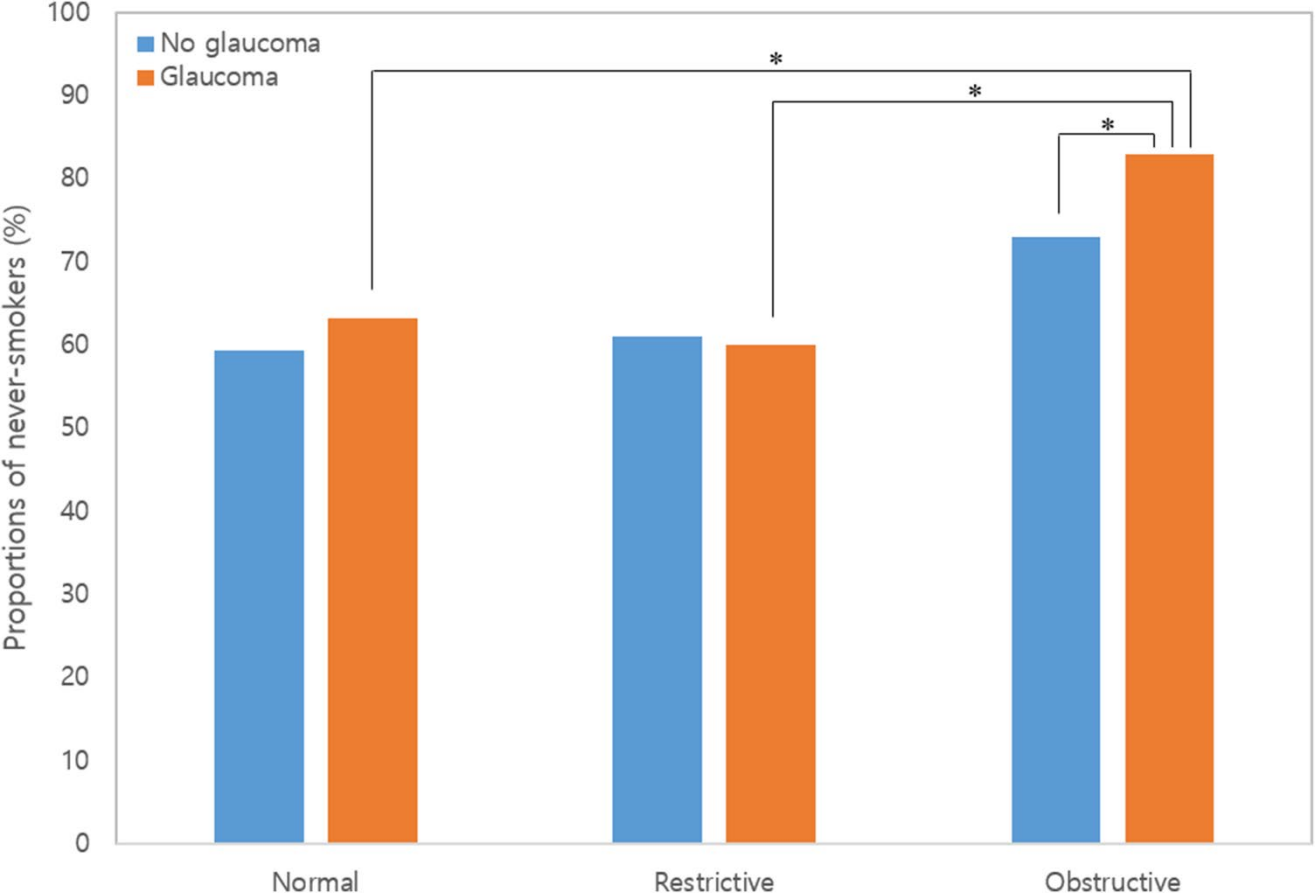
Comparisons of never-smoker proportions in women based on pulmonary function types and the presence of glaucoma. The proportions of never-smokers were significantly higher in women with COPD and glaucoma when compared to women with COPD but no glaucoma. The differences were also statistically significant when compared to women with glaucoma who had restrictive or normal lung function. The asterisk indicates a P value < 0.05.

The three groups were further divided into glaucoma and no glaucoma groups for comparisons. When the proportions of never-smokers in women were analyzed (Fig. 3), the normal and restrictive groups showed similar distribution of never-smokers between those with and without glaucoma (63.2% glaucoma vs. 59.4% no glaucoma, normal; 60.0% glaucoma vs. 61.0% no glaucoma, restrictive). However, in the obstructive group, never-smokers made up the majority of those with glaucoma, and the difference against women without glaucoma was statistically significant (82.6% vs. 73.6%, P = 0.005). The difference was found to be significant when the proportions were compared against women with glaucoma and restrictive lung function, as well as against those women with glaucoma and normal lung function (P < 0.001).

### Association between OAG, smoking status and obstructive pulmonary disease in women

Logistic regression analyses were performed to identify associations between obstructive pulmonary disease and OAG (Table 2). Other than COPD, variables included in the analysis were the same parameters that were found to be significant risk factors of OAG from a previous study that employed the same data set of KNHANES^10^. The analyses revealed that COPD was a significant risk factor for OAG in women when age, IOP, refractive status, BMI, HTN, smoking and exercise (OR 2.077, 95% CI 1.137–3.795, P = 0.017) were controlled. Further analyses were conducted to find if smoking status affected the relationship between COPD and OAG (Table 3). While being a never-smoker alone was not associated with the risk of glaucoma, being a never-smoker with COPD more than doubled the risk of OAG (OR 2.711, 95% CI 1.405–5.232, P = 0.003) in women.

**Table 2:** Logistic regression analysis to identify association between obstructive pulmonary disease and glaucoma in women.

	Model 1			Model 2			Model 3			Model 4		
	OR	95% CI	P	OR	95% CI	P	OR	95% CI	P	OR	95% CI	P
COPD	2.551	1.388–4.688	**0.003**	2.103	1.148–3.851	**0.016**	2.646	1.142–3.845	**0.017**	2.077	1.137–3.795	**0.017**

Model 1 univariate analysis; Model 2 multivariate analysis adjusted for age, refractive status, IOP and BMI; Model 3 multivariate analysis adjusted for age, refractive status, IOP, BMI and HTN; Model 4, multivariate analysis adjusted for age, refractive status, IOP, BMI, HTN, smoking status and exercise.

*OR* odds ratio, *CI* confidence interval, *BMI* body mass index, *COPD* chronic obstructive lung disease.

Significant values are in bold.

A P value < 0.05 was considered statistically significant.

**Table 3:** Logistic regression analysis to identify association between obstructive pulmonary disease, smoking status and glaucoma in women.

	Model 1			Model 2		
	OR	95% CI	P	OR	95% CI	P
COPD	2.551	1.388–4.688	**0.003**	2.646	1.142–3.845	**0.017**
Never smoker	1.110	0.787–1.564	0.553	1.302	0.862–1.966	0.209
COPD x never-smoker	3.457	1.773–6.740	** < 0.001**	2.711	1.405–5.232	**0.003**

Model 1 univariate analysis; Model 2 multivariate analysis adjusted for age, refractive status, IOP, BMI and HTN.

*OR* odds ratio, *CI* confidence interval, *COPD* chronic obstructive pulmonary disease.

Significant values are in bold.

A P-value < 0.05 was considered statistically significant.

## Discussion

In this study, we assessed nationwide, population-based data to identify association between pulmonary function and OAG. Our results demonstrated that OAG was more prevalent in women with COPD than those with restrictive or normal lung function despite similar IOP. COPD was found to increase the risk of OAG in women, and women with COPD were at even higher risks for OAG if they were never-smokers. To the best of our knowledge, our study is the first to identify non-smoking COPD as a risk factor for OAG in Korean women.

Literature on the association between COPD and glaucoma is scarce. The few existing studies have investigated the link between the two diseases in the context of adverse effects of medications. For instance, Huerta et al. found that the prevalence of airway disease was similar between the glaucoma cohort and the general population despite the risk of systemic absorption of beta-blocker eye drops^11^. Another example is continuous and high-dose inhaled steroid, which has long been suspected to increase the risk of secondary glaucoma. A case–control study using the Quebec provincial health insurance plan concluded that the medication did not raise the risk of glaucoma or IOP^12^. However, another study argued that higher doses and longer duration of inhaled corticosteroid for COPD were associated with a higher prevalence of glaucoma^13^. To date, no studies have identified COPD, the disease itself, as an independent risk factor for OAG. We rule out the possibility of COPD medication affecting the prevalence of glaucoma in our study population as none of the women with glaucoma were on COPD medication prior to the spirometry test. There was, however, one man who were on COPD medication and another man with a previous history of COPD treatment at the time of the survey.

Factors that have been postulated to play important roles in the pathogenesis of OAG, in addition to high IOP, include alterations in nitric oxide and oxidative damage^14–16^, hemodynamic abnormality and vascular factors^17^. To that end, previous studies have implicated the role of COPD in propagating both oxidative stress and hemodynamic abnormality. For example, COPD is believed to share common risk factors as well as pathophysiological processes with cardiovascular diseases^18–20^. Based on these findings, it is possible that COPD affects the prevalence of OAG through hemodynamic abnormality. There is also evidence for oxidative stress being a link between the two diseases. Lee et al. analyzed the KNHNAES database and concluded that women with obstructive lung disease had significantly higher serum ferritin levels than women with normal spirometric results^21^. A recent study that also analyzed the KNHANES data set demonstrated that serum ferritin is significantly greater in patients with glaucoma in comparison to control^22^. The authors argued that their findings are associated with iron-related oxidative stress in OAG. These studies suggest that the accumulation of iron can cause degenerative damage in lungs as well as optic nerves^23–26^. Although more extensive studies are needed, oxidative stress, including that caused by ferritin, may be another possible mechanism for the association between COPD and glaucoma.

Our study has identified women as a unique cohort that is substantially affected by the presence of COPD. In both COPD and OAG, women are increasingly found to experience a different course of disease in comparison to their male counterpart. For instance, women with COPD experience more frequent exacerbations^27^, and acute exacerbations are thought to be followed by a period during which the likelihood of cardiovascular events is especially high^28^. Women with COPD show a greater degree of local inflammatory response in the airways^29^, and display higher levels of pro-inflammatory cytokines such as interleukin-16 in comparison to men^30^. We speculate that such differences in the disease course for women may be associated with the differences in distribution of OAG depending on pulmonary function between men and women as demonstrated in our study.

According to the results of our study, having COPD increased the risk of OAG, but being a non-smoker with COPD increased the risk even more. Studies in the past couple of decades has suggested that non-smoking COPD is more prevalent than previously assumed^31^. The pooled results of NHANES reported the prevalence of COPD in never-smokers to be 6.6%^32^. A growing number of investigations have identified traits that distinguish non-smoker COPD from COPD, and these characteristics may explain why non-smoker COPD is more particularly associated with increased prevalence of OAG. For example, non-smoker COPD has shown higher rates of dyspnea and lower oxygen saturation than smoker COPD^33^. Also, acute exacerbations are believed to be more frequent in non-smoker COPD^34^. As mentioned above, the likelihood of cardiovascular events increases following acute exacerbations. During the episodes of exacerbations, more severe oxidative stress has been observed^35^. Lastly, non-smoker COPD is believed to be predominantly female^36,37^.

The effect of lung function on IOP has been investigated, but limited in scope. Variations in pleural pressure during spontaneous breathing in healthy subjects are believed to alter the volume of vascular beds of eyes^38^. As far as intrathoracic pressure is concerned, the pressure is expected to rise in response to limited expiratory air flow in COPD, which may then decrease venous return from the head and elevate IOP in turn. In this regard, the results of comparison of IOP between the three groups was unexpected (Fig. 2). Our analysis showed that the mean IOP of women with obstructive lung function was lower than women with either normal or restrictive lung functions. The mechanism behind lower IOP in women with obstructive lung function is uncertain. We believe that the unanticipated results of our study are the proof that the effect of chronic lung disease on IOP in the long-term is different from that of transient changes in healthy individuals and that studies on the relationship between lung function and IOP are insufficient. Previous studies have revealed that abnormal lung-heart interaction triggers changes in sympathetic system^39,40^, pulmonary and arterial reflexes, and it is possible that such altered relationships in COPD have unidentified effects on IOP. However, further in-depth studies are needed to describe how IOP and optic nerve respond to such complex interactions.

There are several limitations to this study. First, as our study was designed as a cross-sectional analysis, the results only support a significant association between the two diseases. Second, KNHANES utilized FDT instead of automated perimetric tests to assess visual field defects. Although FDT is ideal for large scale screenings, we suspect that the use of FDT may have resulted in overestimation of the number of individuals with glaucoma. In order to minimize overestimation, we excluded participants with a history of retinal disease and stroke, but it might have unintentionally excluded smokers, resulting in exaggerated proportions of never-smokers among those with COPD. Third, the number of individuals diagnosed with glaucoma by the ISGEO criteria were small in each group and may have affected the accuracy of the results. Lastly, the participants of this nationwide survey consist of a homogenous Asian population, among whom NTG is most prevalent. Therefore, cautions are advised when extrapolating these results to other populations.

In conclusion, the nationwide survey of a representative sample of Korean individuals showed that COPD increased the risk of OAG in women. The risk was even higher if COPD was present in never-smokers. Future prospective studies are necessary to confirm the role of COPD in the development of OAG and the mechanism with which the lung function affects the pathogenesis of glaucoma.

## Methods

### Study population

All analyses of this study were conducted on data obtained from KNHANES between 2008 and 2011. KNHANES is a cross-sectional, nationwide, population-based health examinations and survey that is carried out by the Korea Centers for Disease Control and Prevention with the approval from its Institutional Review Board. The study uses a stratified, multi-stage, clustered probability design to select a representative sample of non-institutionalized civilians among the Korean population. The study adhered to the Declaration of Helsinki and all involved participants provided written informed consent. The study consisted of health interviews, health behavior and nutrition survey and health examinations.

A total of 37,753 subjects were included in the KNAHNES conducted between 2008 and 2011. Individuals 40 years or older were included in the analysis (Fig. 1). Participants with a self-reported history of stroke or retinal diseases were excluded from the analysis in order to minimize the risk of including visual field defects not associated with glaucoma. Retinal lesions were also excluded. Participants with shallow anterior chamber (as identified with gonioscopy during examinations) or signs of keratic precipitates were excluded. Those with a history of glaucoma treatment were excluded from the study in order to first eliminate possible effect of β-blocker and prostaglandins on the results of PFT, and second to identify possible differences in treatment-naïve IOP depending on pulmonary function. When both eyes were eligible for inclusion, the right eye was selected. A total of 8941 individuals (3902 men, 5039 women) were subjected to analyses for this study. The study protocol was approved by the Institutional Review Board of Yonsei University Severance Hospital (Approval Number 4-2021-0654).

### Ophthalmic survey

The protocol of ophthalmic examinations for the KNHANES has been described elsewhere^41^. In short, participants underwent visual acuity measurements, automated refraction, slit lamp examination, IOP measurement by trained ophthalmologists using a Goldmann applanation tonometer (Haag-Streit model BQ-900; Haag-Streit, Inc., Bern, Switzerland) and digital fundus photography (TRC-NW6S; Topcon, Inc., Tokyo, Japan, and Nikon, Inc., Tokyo, Japan). Peripheral anterior chamber depth was graded based on Van Herick’s criteria. Vertical cup-to-disc ratios (CDRs) and horizontal CDRs were determined based on the fundus photo images. Spherical equivalent refractive error was calculated as sphere + 1/2 cylinder. Participants additionally underwent visual field examination with frequency doubling technology (FDT) when they met any of the following criteria: (1) IOP ≥ 22, (2) horizontal or vertical CDRs ≥ 0.5, (3) violation of the ISNT rule, (4) optic disc hemorrhage, or (5) retinal nerve fiber layer (RNFL) defects. The FDT test was repeated if fixation errors or false-positive responses were greater than 33%. If a participant failed to respond below the 33% mark in the second test, the test was considered invalid. The diagnosis of glaucoma was made according to the modified International Society of Geographical and Epidemiological Ophthalmology (ISGEO) criteria. Category 1 required both a reliable visual field defect and glaucomatous optic disc (1) neuroretinal rim loss with vertical or horizontal CDR ≥ 0.6, (2) optic disc hemorrhage, (3) RNFL defects, or (4) asymmetry in vertical CDR ≥ 0.2). When FDT results were invalid or unavailable, category 2 was applied: (1) vertical CDR ≥ 0.9 (99.5th percentile), or (2) asymmetry of vertical CDR ≥ 0.3 (99.5th percentile). A defect was considered present if two different test points were abnormal.

### Spirometric tests

Spirometry (Vmax 2130 Dry Rolling-seal Spirometer; SensorMedics, Yorba Linda, CA, USA) was conducted by four trained technicians. The test protocol followed the guidelines outlined by the American Thoracic Society/European Respiratory Society for standardizing the pulmonary function tests^42^. The tests that produced two acceptable spirometry curves (defined as curves which show the start of the test and expiration ≥ 6 s, show greatest differences between the two measurements of forced expiratory volume in 1 s (FEV_1_) and forced vital capacity (FVC) < 150 mL) were considered valid and included for the analysis. Participants were considered to have COPD if the ratio of FEV_1_ to FVC (FEV_1_/FVC) was below 70% according to the Global Initiative for Chronic Obstructive Lung Disease^43^. A restrictive lung disease was diagnosed if FEV_1_/FVC ≥ 70% and FVC was < 80% predicted. Subjects were considered to have normal lung function if FEV_1_/FVC ≥ 70% and FVC ≥ 80% predicted.

### Statistical analysis

Statistical analyses were performed using SPSS statistical software (version 23; IBM Corporation, Armonk, NY). The PROC SURVEY procedure with sample weights was used to provide nationally representative prevalence estimates as recommended by the KNHANES, which employs complex sampling. The continuous variables were presented as mean (standard deviation) and the categorical variables were presented as number (percentage). Comparisons between groups were made using the Rao-Scott chi-square test (for categorical variables) or the Wald test (for continuous variables) as recommended. Simple and multivariate logistic regression analyses were conducted to identify association between the prevalence of OAG and obstructive lung disease. Participants with restrictive patterns were added to the normal group for regression analyses. The multivariate logistic regression model included as potential confounding variables those variables found to be significant risk factors of OAG from a previous study that analyzed the same data set of KNHANES, namely age, gender, IOP, myopia, hypertension and BMI^10^. Odds ratios (OR) and 95% confidence interval (CI) were calculated. For all analyses, the P-values were two-tailed and values < 0.05 were considered statistically significant.

## Data Availability

The data that support the findings of this study are available in Korean National Health and Nutrition Examination Surveys at https://knhanes.kdca.go.kr/knhanes/main.do.

## References

[1] Pascolini D (2004). 2002 global update of available data on visual impairment: A compilation of population-based prevalence studies. Ophthalmic Epidemiol..

[2] Quigley HA, Broman AT (2006). The number of people with glaucoma worldwide in 2010 and 2020. Br. J. Ophthalmol..

[3] Miglior S (2005). Results of the European glaucoma prevention study. Ophthalmology.

[4] Leske MC (2008). Risk factors for incident open-angle glaucoma: The Barbados eye studies. Ophthalmology.

[5] Fan YY (2019). Correlation between structural progression in glaucoma and obstructive sleep apnea. Eye.

[6] Lei X (2019). Choroidal detachment and increased intraocular pressure in a case of secondary pulmonary hypertension. J. Glaucoma.

[7] Senthil S, Kaur B, Jalali S, Garudadri C (2009). Secondary open-angle glaucoma and central retinal vein occlusion in a patient with primary pulmonary hypertension. Ophthalmic Surg. Lasers Imaging.

[8] Falk JA (2008). Cardiac disease in chronic obstructive pulmonary disease. Proc. Am. Thorac. Soc..

[9] Dursunoglu N (2017). Severity of coronary atherosclerosis in patients with COPD. Clin. Respir. J..

[10] Kim KE (2016). Prevalence, awareness and risk factors of primary open-angle glaucoma: Korea national health and nutrition examination survey 2008–2011. Ophthalmology.

[11] Huerta C, Garcia Rodriguez LA, Moller CS, Arellano FM (2001). The risk of obstructive airways disease in a glaucoma population. Pharmacoepidemiol. Drug Saf..

[12] Gonzalez AV, Li G, Suissa S, Ernst P (2010). Risk of glaucoma in elderly patients treated with inhaled corticosteroids for chronic airflow obstruction. Pulm. Pharmacol. Ther..

[13] Nath T (2017). Prevalence of steroid-induced cataract and glaucoma in chronic obstructive pulmonary disease patients attending a tertiary care center in India. Asia Pac. J. Ophthalmol..

[14] Shen F (2004). Glutamate-induced glutamine synthetase expression in retinal Muller cells after short-term ocular hypertension in the rat. Investig. Ophthalmol. Vis. Sci..

[15] Galassi F (2004). Nitric oxide proxies and ocular perfusion pressure in primary open angle glaucoma. Br. J. Ophthalmol..

[16] Chung HS (1999). Vascular aspects in the pathophysiology of glaucomatous optic neuropathy. Surv. Ophthalmol..

[17] Lee SH (2017). Vascular and metabolic comorbidities in open-angle glaucoma with low- and high-teen intraocular pressure: A cross-sectional study from South Korea. Acta Ophthalmol..

[18] Baum C (2016). Subclinical impairment of lung function is related to mild cardiac dysfunction and manifest heart failure in the general population. Int. J. Cardiol..

[19] Lipworth B (2016). Underuse of beta-blockers in heart failure and chronic obstructive pulmonary disease. Heart.

[20] Vestbo J (2016). Fluticasone furoate and vilanterol and survival in chronic obstructive pulmonary disease with heightened cardiovascular risk (SUMMIT): A double-blind randomised controlled trial. Lancet.

[21] Lee CH (2016). Association of serum ferritin levels with smoking and lung function in the Korean adult population: Analysis of the fourth and fifth Korean national health and nutrition examination survey. Int. J. Chron. Obstr. Pulm. Dis..

[22] Lin SC, Wang SY, Yoo C, Singh K, Lin SC (2014). Association between serum ferritin and glaucoma in the South Korean population. JAMA Ophthalmol..

[23] Chen H (2009). Changes in iron-regulatory proteins in the aged rodent neural retina. Neurobiol. Aging.

[24] Aquino D (2009). Age-related iron deposition in the basal ganglia: Quantitative analysis in healthy subjects. Radiology.

[25] Altamura S, Muckenthaler MU (2009). Iron toxicity in diseases of aging: Alzheimer’s disease, Parkinson’s disease and atherosclerosis. J. Alzheimers Dis..

[26] Kell DB (2009). Iron behaving badly: Inappropriate iron chelation as a major contributor to the aetiology of vascular and other progressive inflammatory and degenerative diseases. BMC Med. Genom..

[27] de Torres JP, Casanova C, Montejo de Garcini A, Aguirre-Jaime A, Celli BR (2007). Gender and respiratory factors associated with dyspnea in chronic obstructive pulmonary disease. Respir. Res..

[28] Donaldson GC, Hurst JR, Smith CJ, Hubbard RB, Wedzicha JA (2010). Increased risk of myocardial infarction and stroke following exacerbation of COPD. Chest.

[29] Gut-Gobert C (2019). Women and COPD: Do we need more evidence?. Eur. Respir. Rev..

[30] de Torres JP (2011). Gender differences in plasma biomarker levels in a cohort of COPD patients: A pilot study. PLoS ONE.

[31] Salvi SS, Barnes PJ (2009). Chronic obstructive pulmonary disease in non-smokers. Lancet.

[32] Behrendt CE (2005). Mild and moderate-to-severe COPD in nonsmokers: Distinct demographic profiles. Chest.

[33] Meneghini AC (2019). Biomass smoke COPD has less tomographic abnormalities but worse hypoxemia compared with tobacco COPD. Braz. J. Med. Biol. Res..

[34] Jindal SK (2020). COPD exacerbation rates are higher in non-smoker patients in India. Int. J. Tuberc. Lung Dis..

[35] Drost EM (2005). Oxidative stress and airway inflammation in severe exacerbations of COPD. Thorax.

[36] Buist AS (2007). International variation in the prevalence of COPD (the BOLD Study): A population-based prevalence study. Lancet.

[37] Bridevaux PO (2010). Prevalence of airflow obstruction in smokers and never-smokers in Switzerland. Eur. Respir. J..

[38] Lundmark PO, Trope GE, Flanagan JG (2003). The effect of simulated obstructive apnoea on intraocular pressure and pulsatile ocular blood flow in healthy young adults. Br. J. Ophthalmol..

[39] Forfia PR, Vaidya A, Wiegers SE (2013). Pulmonary heart disease: The heart-lung interaction and its impact on patient phenotypes. Pulm. Circ..

[40] Andre S (2019). COPD and cardiovascular disease. Pulmonology.

[41] Lee SH (2016). Estimated trans-lamina cribrosa pressure differences in low-teen and high-teen intraocular pressure normal tension glaucoma: The Korean national health and nutrition examination survey. PLoS ONE.

[42] Miller MR (2005). Standardisation of spirometry. Eur. Respir. J..

[43] Vestbo J (2013). Global strategy for the diagnosis, management and prevention of chronic obstructive pulmonary disease: GOLD executive summary. Am. J. Respir. Crit. Care Med..

